# Supplementary material for: Revisiting IgG Antibody Reactivity to Epstein-Barr Virus in Myalgic Encephalomyelitis/Chronic Fatigue Syndrome and Its Potential Application to Disease Diagnosis
Source: Front Med (Lausanne). 2022 Jun 24;9:921101. doi: 10.3389/fmed.2022.921101 (PMC9263839; doi:10.3389/fmed.2022.921101)

Supplementary Table 1

The overall and per-EBV-strain number of 15-mer peptides (antigens) whose antibody responses analysed.

EBV Protein	Associated stage	Number of 15-mer peptides per EBV strain						
		Overall	AG876	B95.8	GD1	Cao	Raji	P3HR.1
BALF-2	Early lytic	290	278	278	278	0	0	0
BALF-5	Early lytic	256	250	250	250	0	0	0
BFRF-3	Late lytic	42	0	42	0	0	0	0
BLLF-1	Late lytic	273	204	202	199	0	0	204
BLLF-3	Early lytic	74	66	67	66	0	0	0
BLRF-2	Late lytic	41	38	38	38	0	0	0
BMRF-1	Early lytic	102	99	99	99	0	0	0
BZLF-1	Immediate early lytic	89	57	57	58	0	0	0
EBNA-1	Latency I, II, and III	182	98	107	111	0	0	0
EBNA-3	Latency III	446	223	226	224	0	0	0
EBNA-4	Latency III	469	229	221	224	0	0	0
EBNA-6	Latency III	461	254	234	230	0	0	0
LMP-1	Latency II and III	197	79	85	80	77	84	0
LMP-2	Latency II and III	132	120	120	120	0	0	0

Supplementary Table 2

Comparison among different null models (including the covariates age and gender and their interaction) using the Akaike's information criterion (AIC). The best model for each analysis/comparison is shown in bold. ME/CFS_all, ME/CFS_inf and ME/CFS_noninf represent all the ME/CFS patients, ME/CFS patients with an infectious trigger, and ME/CFS patients with a non-infectious trigger, respectively.

Analysis/Comparison	Model (Link function)	AIC	ROC (95% CI)
ME/CFS_all vs Healthy Controls	Logit	189.973	0.577 (0.478;0.676)
	Probit	189.964	0.576 (0.478;0.675)
	**Complementary log-log**	**189.936**	**0.574 (0.475;0.672)**
ME/CFS_inf vs Healthy Controls	Logit	147.055	0.610 (0.500;0.719)
	**Probit**	**147.029**	**0.606 (0.496;0.715)**
	Complementary log-log	147.220	0.609 (0.499;0.718)
ME/CFS_noninf vs Healthy Controls	Logit	127.619	0.556 (0.429;0.683)
	Probit	127.629	0.559 (0.432;0.687)
	**Complementary log-log**	**127.547**	**0.556 (0.429;0.683)**
ME/CFS_inf vs ME/CFS_noninf	**Logit**	**129.205**	**0.596 (0.471;0.720)**
	Probit	129.236	0.597 (0.472;0.721)
	Complementary log-log	129.529	0.596 (0.472;0.721)

Supplementary Table 3

The top 5 most significant antibodies for each association analysis where ME/CFS_all, ME/CFS_inf and ME/CFS_noninf represent all ME/CFS patients, ME/CFS patients with an infectious trigger, and ME/CFS patients with a non-infectious trigger, respectively. For simplicity, the antibodies were identified by their peptide. Statistically significant findings were obtained for $-\log_{10}$(adjusted p-value) > 1.30 (=$-\log_{10}$(0.05)) controlling for false discovery rate of 5% using the Benjamini-Yekutieli procedure.

**Analysis/Comparison**	**Peptide**	**$-\log_{10}$(adjusted p-value)**
ME/CFS_all vs Healthy controls	EBNA6_0066	0.743
	BLRF2_0005	0.486
	EBNA4_0392	0.486
	EBNA4_0497	0.486
	EBNA4_0529	0.486
ME/CFS_inf vs Healthy controls	EBNA6_0066	2.693
	EBNA6_0070	2.693
	EBNA4_0529	1.794
	EBNA3_0380	1.270
	EBNA6_0569	1.270
ME/CFS_noninf vs Healthy controls	EBNA6_0782	1.193
	BALF2_0358	1.153
	BALF2_0765	1.153
	BALF5_0041	1.153
	BALF5_0206	1.153

Supplementary Figure 1

Distributions of the Spearman's correlation coefficient between all the possible pairs of EBV-derived antibodies in healthy controls, all the ME/CFS patients, ME/CFS patients with an infectious trigger, and ME/CFS patients with a non-infectious or unknown trigger.

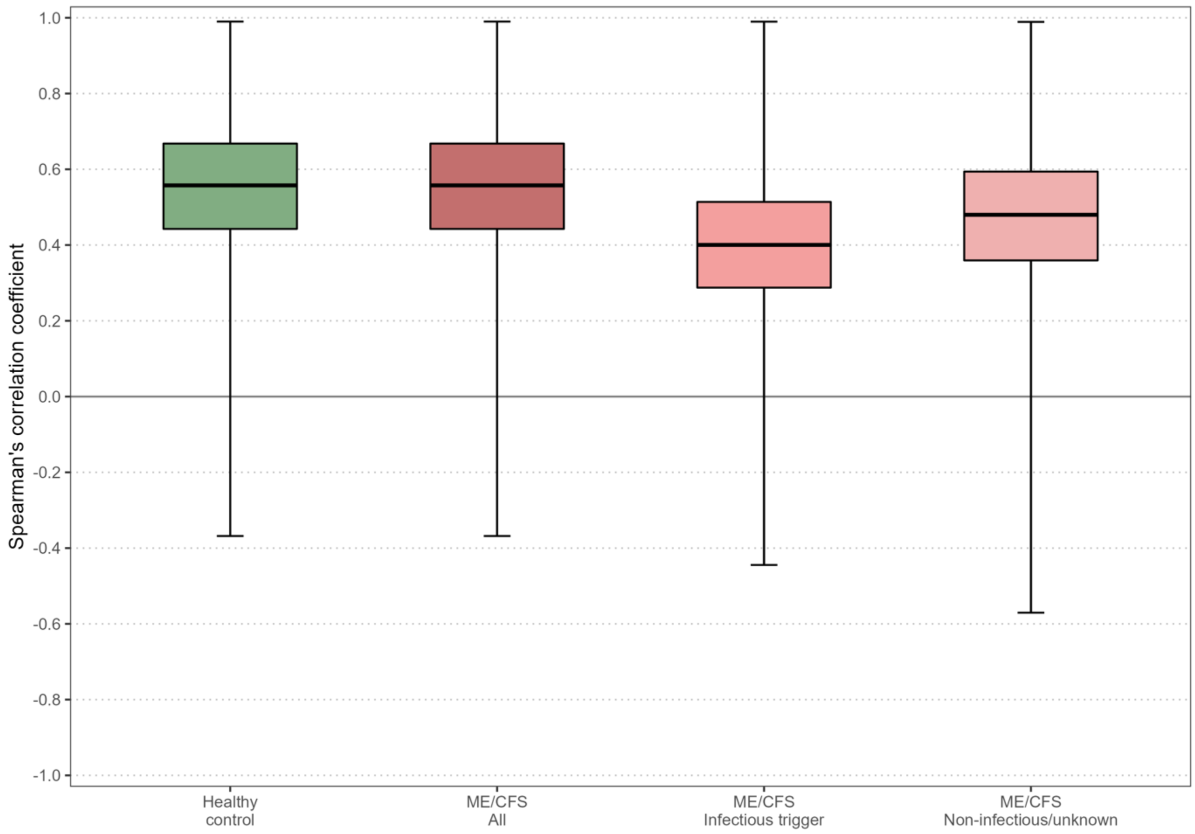

Supplement: Supplementary file 1 [file Data_Sheet_1.pdf]
